# Breast Cancer Classification Using Synthesized Deep Learning Model with Metaheuristic Optimization Algorithm

**DOI:** 10.3390/diagnostics13182925

**Published:** 2023-09-12

**Authors:** Selvakumar Thirumalaisamy, Kamaleshwar Thangavilou, Hariharan Rajadurai, Oumaima Saidani, Nazik Alturki, Sandeep kumar Mathivanan, Prabhu Jayagopal, Saikat Gochhait

**Affiliations:** 1 Department of Artificial intelligence & Data Science, Dr. Mahalingam College of Engineering and Technology, Pollachi 642003, India; 2 Department of Computer Science and Engineering, Vel Tech Rangarajan Dr. Sagunthala R&D Institute of Science and Technology, Chennai 600062, India; 3 School of Computing Science and Engineering, VIT Bhopal University, Bhopal–Indore Highway Kothrikalan, Sehore 466114, India; 4 Department of Information Systems, College of Computer and Information Sciences, Princess Nourah bint Abdulrahman University, P.O. Box 84428, Riyadh 11671, Saudi Arabia; 5 School of Computing Science & Engineering, Galgotias University, Greater Noida 203201, India; 6 School of Computer Science Engineering and Information Systems, Vellore Institute of Technology, Vellore 632014, India; jprabhuit@gmail.com; 7 Symbiosis Institute of Digital and Telecom Management, Constituent of Symbiosis International Deemed University, Pune 412115, India; 8 Neuroscience Research Institute, Samara State Medical University, 443001 Samara, Russia

**Keywords:** transfer learning, breast cancer, convolutional neural network, Ant Colony Optimization, ResNet101, hyperparameters

## Abstract

Breast cancer is the second leading cause of mortality among women. Early and accurate detection plays a crucial role in lowering its mortality rate. Timely detection and classification of breast cancer enable the most effective treatment. Convolutional neural networks (CNNs) have significantly improved the accuracy of tumor detection and classification in medical imaging compared to traditional methods. This study proposes a comprehensive classification technique for identifying breast cancer, utilizing a synthesized CNN, an enhanced optimization algorithm, and transfer learning. The primary goal is to assist radiologists in rapidly identifying anomalies. To overcome inherent limitations, we modified the Ant Colony Optimization (ACO) technique with opposition-based learning (OBL). The Enhanced Ant Colony Optimization (EACO) methodology was then employed to determine the optimal hyperparameter values for the CNN architecture. Our proposed framework combines the Residual Network-101 (ResNet101) CNN architecture with the EACO algorithm, resulting in a new model dubbed EACO–ResNet101. Experimental analysis was conducted on the MIAS and DDSM (CBIS-DDSM) mammographic datasets. Compared to conventional methods, our proposed model achieved an impressive accuracy of 98.63%, sensitivity of 98.76%, and specificity of 98.89% on the CBIS-DDSM dataset. On the MIAS dataset, the proposed model achieved a classification accuracy of 99.15%, a sensitivity of 97.86%, and a specificity of 98.88%. These results demonstrate the superiority of the proposed EACO–ResNet101 over current methodologies.

## 1. Introduction

Breast cancer is a major global health issue and one of the most frequent cancers in women worldwide, with greater rates in developing nations, according to the WHO. It causes 11.7% of all female cancer deaths [1]. Early breast cancer identification improves therapy and results. Neglecting early detection may spread cancer to other parts of the breast or body, making treatment harder and limiting survival [2]. Various screening techniques, such as mammography and breast self-examination, play crucial roles in detecting breast cancer at its early stages. Medical imaging has witnessed significant advances, with convolutional neural networks (CNNs) showing promise in improving the accuracy of tumor detection and classification [3]. Traditional methods of classifying breast cancer have struggled to keep up with the rising demand for medical images, posing challenges to public health. However, the development and extensive use of various imaging techniques have facilitated early breast cancer detection [4].

Mammography, breast ultrasonography, MRI, PET, and CT can all be used to identify breast cancer. Breast ultrasonography serves both diagnostic and therapeutic purposes. Therapeutic ultrasound treats without imaging, whereas diagnostic ultrasound provides images [5]. Tumors are typically classified as benign or malignant. Although benign tumors are not life-threatening, they can increase the risk of breast cancer in women [6]. Malignant tumors, on the other hand, are cancerous and require immediate attention. A study on breast cancer screening revealed that 20% of women had cancerous tumors [7]. Improving cancer detection and prognosis is crucial for ensuring better patient outcomes and survival rates. Consequently, machine learning (ML) techniques have emerged as a research priority for early breast cancer diagnosis and prognosis [8]. This study utilized SVM, random forest, logistic regression, decision tree (C4.5), and KNN classifiers to assess and compare their performance for breast cancer diagnosis and prognosis [9].

The primary goal of this research is to determine the most effective ML (ML) algorithms for accurate breast cancer predictions and diagnoses. Among the classifiers tested, support vector machines (SVMs) exhibited the highest accuracy at 97.2% [10]. The author utilized a number of popular machine learning (ML) methods throughout the inquiry phase, including RF, DT, KNN, and logistic regression, with corresponding F1 scores of 96%, 95%, 90%, and 98%. On Google Colab, the average algorithm execution time was between two and three minutes. This investigation relied on mathematical models, the accuracy of which was substantially greater than in earlier studies [11]. The author of this piece developed a method for optimizing algorithms automatically in the field of CAD. Five ML classifiers were used to determine whether tumors were malignant or benign, and ML was trained using 13 of the 185 characteristics. Experimental findings showed that the best results were achieved by combining a tree-structured Parzen estimator with an ML classifier, and a 10-fold cross-validation Bayesian optimization [12]. Among the four classifiers used, the LightGBM classifier performed the best, achieving an accuracy of 99.86%, precision of 100%, recall of 99.60%, and F1 score of 99.80% [13]. By using data exploratory techniques (DET), the author created four alternative prediction models to improve the accuracy of breast cancer evaluation. In order to effectively classify features into malignant and benign classifications, a thorough assessment of four-layered essential dimensionality reduction technique (DET) processes was performed before modeling [14]. These procedures included feature distribution, correlation analysis, feature removal, and hyperparameter optimization. The effectiveness of the suggested methods and classifiers was tested using the Wisconsin Diagnostic Breast Cancer (WDBC) and the Breast Cancer Coimbra Dataset (BCCD). Classifiers’ efficacy and training times were measured using common performance measures, including confusion matrices and K-fold cross-validation. Our DET method significantly improved the models’ diagnostic performance. The polynomial SVM achieved 99.3% accuracy on the WDBC dataset, followed by LR with 98.06%, KNN with 97.35%, and EC with 97.61% [15].

In this work, the author employed a technique known as hybrid ML systems (HMLS) to identify breast cancer. The primary objective of this work is to identify a prioritized transcriptome pattern that can be linked to classification tools for early breast cancer diagnosis. To find the best HMLS, this method utilizes feature selection algorithms, a feature extraction methodology, and classifiers. The logistic regression plus logistic regression (LGR + LR) classifier ranked as the second best among the employed feature selection strategies in terms of prediction accuracy and AUC (area under the curve). The overall accuracy was 0.86, and the area under the curve (AUC) was 0.94. Furthermore, a classifier based on LGR and MLP performed well, achieving an AUC of 0.94 and a balanced accuracy of 0.84 [16].

The purpose of this research was to evaluate the performance of ML-based estimations in comparison to two widely used methods: the Breast Cancer Risk Assessment Tool (BCRAT) and the Breast and Ovarian Analysis of Disease Incidence and Carrier Estimation Algorithm (BOADICEA). Both ML–adaptive boosting and ML–random forest achieved a prediction accuracy (AU-ROC curve) of 88.28% for the U.S. population-based sample, while the BCRAT approach only reached 62.40% accuracy [17]. In the Swiss clinical sample, the prediction accuracy of ML adaptive boosting was 90.1%, while BOADICEA achieved only 59.31% accuracy. An accuracy of 89.32% was also achieved [18] using an ML–Markov chain Monte Carlo generalized linear mixed model. The primary goal of this research was to evaluate ML-based estimates in terms of their discriminating accuracy in comparison to BOADICEA and BCRAT. Both the U.S. population-based sample and the Swiss clinical sample demonstrated that ML-based techniques, especially ML–adaptive boosting and ML–random forest, outperformed the conventional methods. These ML models had far greater predicted accuracies than the BOADICEA and BCRAT models [19].

The author conducted studies using ML techniques such as convolutional neural networks (CNN), support vector machines (SVM), and random forests (RF) to diagnose breast cancer. The objective was to compare the performance of these various approaches. The results showed that, in terms of accuracy, precision, and data utilization, the CNN method was superior to SVM and RF. Specifically, when compared to SVM (89.84%) and RF (90.55%), CNN achieved an impressive accuracy of 99.67% [20]. However, it should be noted that another study aimed to improve breast cancer detection accuracy by leveraging hidden data features. Data on demographics, laboratory results, and mammograms were analyzed using various ML methods in this investigation, using records from the Motamed Cancer Institute (ACECR) in Tehran, Iran. Twenty-five percent of the database’s 5178 records represented people with breast cancer, and each record contained 24 fields. With an accuracy of 80%, sensitivity of 95%, specificity of 80%, and an area under the curve (AUC) of 0.56, the RF technique demonstrated superiority over other approaches in this study. The AUC for the gradient boosting (GB) approach was 0.59 [21], which was higher than that of the neural network. This research provides an introductory overview of several deep learning methods, including the artificial neural network (NN), FM deep learning, convolutional NN, and recurrent NN. In this study, we offer a full elucidation of the theory, developmental trajectory, and practical implementations of the aforementioned concepts in the realm of illness prediction. Additionally, we undertake an examination of the existing deficiencies in illness prediction and propose prospective remedies. Moreover, this research examines two significant developments that are influencing the future of illness prediction and the medical field: the incorporation of digital twins and the advancement of precision medicine [22]. This study presents a novel medical diagnostic method that has been developed specifically for the purpose of forecasting the likelihood of cardiovascular illness. The proposed system integrates the evolutionary algorithm and neural network, capitalizing on their respective strengths. In particular, it employs multilayered feed-forward neural networks to address intricate categorization tasks. The utilization of the genetic algorithm is applied as an effective method for determining the weights of the neural network. This algorithm has the capability to identify an appropriate set of weights in a reduced number of iterations. The dataset from the University of California, Irvine (UCI) ML repository is utilized for both training and testing purposes [23]. Deep learning plays a vital role in illness prediction, particularly in the medical field, where there are abundant datasets containing latent patterns. The retrieval of pertinent medical data for educational purposes is crucial. The focus of this study is the application of deep learning techniques for the prediction of fever-related illnesses. The objective of this study is to examine and contrast the duration of evaluation, the accuracy of classification, and the precision in detecting diseases. The empirical findings demonstrate that the categorization performance of deep neural networks surpasses that of existing classification methods, resulting in improved accuracy when assessing the severity of diseases [24]. In order to speed up the diagnostic procedure, the researchers in this work used an adaptive evolutionary algorithm to optimize the parameters for the deep learning model. Three popular deep learning models were modified to incorporate this method, and tests were run using both this method and other well-known ML approaches to determine its efficacy. According to the results of the studies, the suggested model has a higher rate of accuracy than the competing methods, reaching around 96% for multiclass classification and 98% for binary classification [25].

Genetic algorithms play a crucial role as an optimization technique in the field of ML, particularly in the context of classification problems. They are highly regarded for their ability to achieve a notable level of prediction accuracy. In this study, genetic algorithms are utilized to analyze and predict cardiac problems in individuals with coronary heart disease, which is a clinically relevant ailment characterized by the narrowing of the coronary arteries responsible for supplying oxygenated blood to the heart. The heart disease dataset obtained from the UCI ML library is employed to enhance the accuracy of classification and prediction for patients diagnosed with various cardiac diseases [26].

ML methodologies are used to identify significant patterns and make predictions about future occurrences or trends. The objective of this study is to employ a modified ML algorithm to forecast the probability of coronary heart disease occurrence in individuals. The input data undergoes a series of processes, including preprocessing, grouping, and identifying relevant attributes before classification. The study integrates four algorithms, namely random forest, K-means, genetic algorithm, and logistic regression, to ascertain the presence of heart disease. To improve performance and reduce training time, the cardiac dataset is subjected to a feature selection process using random forest. Genetic algorithms are utilized to optimize K-means clustering, facilitating the efficient grouping of outlier data points. Finally, logistic regression is employed to categorize individuals based on the presence of heart disease. The suggested methodology is evaluated against established approaches using several performance metrics, demonstrating a significant improvement in accuracy of up to 95% [27]. The integration of deep learning algorithms with the genetic algorithm has several advantages that capitalize on the respective strengths of each technique. Genetic algorithms have a high level of proficiency in identifying approximate solutions within intricate search spaces, while deep learning algorithms exhibit exceptional aptitude in optimizing intricate models. The integration of both components enhances the optimization procedure, resulting in increased resilience and effectiveness, thus facilitating the identification of superior solutions. An important benefit of this integration is its ability to mitigate the issue of overfitting in deep learning models. Overfitting is a phenomenon that arises when models exhibit high performance on the training data but demonstrate poor generalization ability when applied to unknown data. The incorporation of variation in the model parameters using genetic algorithms can effectively address the issue of overfitting, leading to enhanced generalization capabilities and higher performance when applied to novel data. Another advantage is the accelerated convergence of deep learning training. The process of training deep learning models is characterized by its high computing demands and time-intensive nature. Genetic algorithms have the potential to expedite convergence by furnishing a favorable initial parameter set, thereby enhancing the efficiency of the deep learning model in attaining optimum solutions more rapidly. In addition, deep learning models frequently incorporate several hyperparameters that have a substantial influence on their performance. Genetic algorithms have the ability to effectively explore and optimize hyperparameters, resulting in improved model performance. Moreover, the intrinsic parallelizability of genetic algorithms renders them well-suited for deployment in distributed computing environments. When combined with deep learning, their scalability enables efficient management of extensive datasets and intricate models.

## 2. Preliminaries

### 2.1. Ant Colony Optimization

Ants exhibit communal behavior, and the Ant Colony Optimization (ACO) concept revolves around observing individual ant behavior as they leave their nests and explore various paths to find the best route for locating food. Initially, ants engage in disorganized foraging behavior around their nests as they search for food sources. This stochastic exploration results in several routes connecting the nest and the food supply. The transportation of food by ants depends on both the quantity and quality of the food. As ants traverse the paths, they leave behind a trail of pheromones, creating a concentration suitable for guiding their fellow ants to the food source. The outcomes of these pheromone experiments significantly influence the probability of other ants choosing a particular path to access the food source, making the pheromone trail a crucial guiding determinant. The probability of selecting a specific route is contingent upon the concentration of the pheromone and its rate of evaporation. It is important to note that the duration of each route should also consider the rate of pheromone evaporation [28].

**Figure 1:**
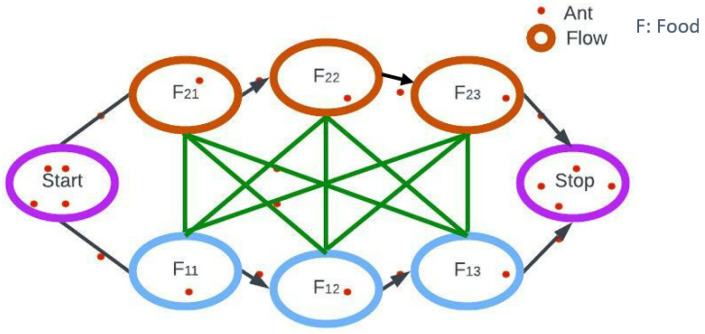
Ant Colony Optimization approach.

To maintain simplification, only two routes between the food source and the ant nest have been taken into account in Figure 1 above. Following is an analysis of the stages.

Stage 1: Every ant is in its nest. The environment has no pheromone content. (Residual pheromone amount can be taken into account for algorithmic design without affecting probability).

Stage 2: Ants start their search along each path with an equal (0.5) probability. It is obvious that the curved path is longer, and as a result, it takes more time for the ants to reach the food source.

Stage 3: The ants travel a shorter distance and arrive at the food source earlier. They are now faced with a similar selection conundrum, but this time, since there is already a pheromone trail along the shorter path, the likelihood of selection is higher.

Stage 4: Pheromone concentrations rise as a result of more ants using the shorter path. Additionally, the pheromone concentration on the longer path decreases due to evaporation, lowering the likelihood that this path will be chosen in later stages. As a result, the entire colony gradually takes the shorter path more frequently. Consequently, path optimization is achieved.

Ants rely on a network of pathways and chemical trails, known as pheromones, to efficiently navigate their environment. During food foraging, ants send out scouts in various directions, and these scouts engage in a stochastic process of path selection while depositing pheromone trails along their movement. As more ants follow these pathways, the pheromone trails intensify, directing subsequent ants toward the food source. Interestingly, ants do not have the ability to assess the length of a path or calculate the time required to reach a food source. Instead, their food-finding efficiency is enhanced through simple behavioral principles that have evolved over time. In the context of a curved trajectory, certain ants may initially choose to follow it due to stochastic selection or detecting a subtle pheromone trail left by a scout ant. However, if this specific route takes significantly more time to reach the food source, the intensity of the pheromone trail left by ants along this route will be comparatively lower than the trail established on a shorter path. Consequently, over time, ants are more likely to choose the shorter route, even if both pathways were initially explored with equal likelihood.

(1) $$A_{i}=\frac{P_{i}}{P_{1}+P_{2}};i=1,2$$

(2) $$P_{i}\leftarrow P_{i}+\frac{R}{K_{i}}$$

(3) $$P_{i}\leftarrow \left(1-u\right)*P_{i}$$

For edges *E*_1_ and *E*_2_, we can suppose that their associated pheromone values (indicating their strength) are *P*_1_ and *P*_2_, respectively. If *P*_1_ is greater than *P*_2_, it stands to reason that the odds of picking *E*_1_ are greater. Now, on the way back along this shortest path, let us call it *E_i_*, the relevant pheromone value is modified.

### 2.2. Opposition-Based Learning (OBL)

Opposition-based learning (OBL) has the potential to improve the accuracy and robustness of ML models, but it is not specifically designed to address challenges related to preventing stagnation in rival solutions or optimizing search mechanism exploitation [29]. Stagnation in rival solutions refers to the use of outdated or inefficient approaches to solving a problem, while search mechanism exploitation involves enhancing the search strategy to efficiently explore the search area. The primary objective of OBL is to optimize the learning process by simultaneously training two models and integrating their respective perspectives. It helps in selecting relevant features and optimizing the model. However, it does not directly tackle issues related to being outdated or abusing search mechanisms. To address these concerns, OBL can be combined with other methodologies, such as meta-learning or reinforcement learning techniques, to dynamically adapt the search strategy or update model parameters in real-time [30]. This integration can enhance overall performance and efficiency, reducing stagnation in rival solutions and improving search mechanism exploitation. In the OBL framework, two models are trained simultaneously: the main model and the opposition model. The opposition model is constructed by negating either the input data, weights, or output of the primary model. During training, both models undergo iterative updates based on each other’s performance. The main model optimizes the objective function in a certain direction, while the opposition model seeks to minimize it in the opposite direction. The answers generated by both models are evaluated, and the optimal course of action is selected. The definition takes into account Z_0_ considered as a real number Є [ø, *q*]. The opposite number of Z_0_ is represented in the Equation (4),

(4) $$\bar{Z_{0}}=\emptyset +q-Z_{0}$$

Calculating the opposite number in dimensional (N) space shown in Equations (5) and (6),

(5) $$Z=Z_{1},Z_{2},Z_{3,}Z_{4},\ldots Z_{N}$$

(6) $$\bar{Z}=[{\bar{Z}}_{1},{\bar{Z}}_{2,}{\bar{Z}}_{3},{\bar{Z}}_{4},\ldots {\bar{Z}}_{N}]$$

Here, the value of Z is demonstrated by Equation number (7),

(7) $${\bar{Z}}_{l}=\emptyset_{r}+q_{r}-Z_{r}$$

where *r* = 1, 2, 3, 4…*N*.

The opposite item $\bar{Z_{0}}$ is changed with the corresponding solution $Z_{0}$ throughout the fitness function. If $f_{t}(Z_{0})$ is better than $f_{t}({\bar{Z}}_{0})$, $Z_{0}$ is considered as constant; then $Z_{0}={\bar{Z}}_{0}$. The solutions have been changed to reflect the best value for Z and $\bar{Z}$ as a result.

### 2.3. Convolutional Neural Network (CNN)

**Figure 2:**
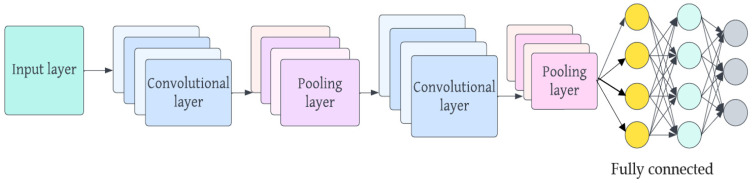
Convolutional neural network architecture.

Convolutional neural networks (CNNs) are a subset of deep neural networks that have found widespread use in image recognition and classification, and their essential components are discussed here. CNNs are built using linked layers of neurons to train on their own and extract information from data. Convolutional layers, pooling layers, and fully linked layers are common components of this layer structure. Convolutional layers use filters to find regional patterns and characteristics by performing convolutions on the input data. To improve translation invariance and decrease the dimensionality of feature maps, pooling layers are used. When neurons in one layer are linked to those in the next, the network can learn more complicated information and perform categorization tasks. In recent years, CNNs have become increasingly popular for use in image analysis [31]. Developed in 1989, CNNs have proven to be quite useful for image segmentation and classification. They take inspiration from the way the human brain processes visual information, with many layers of “neurons” that selectively respond to other neurons in their immediate vicinity. With their convolutional layers, pooling layers, and fully connected layers, CNNs properly capture an image’s topological features. An example of a common CNN architecture is shown in Figure 2.

**Figure 3:**
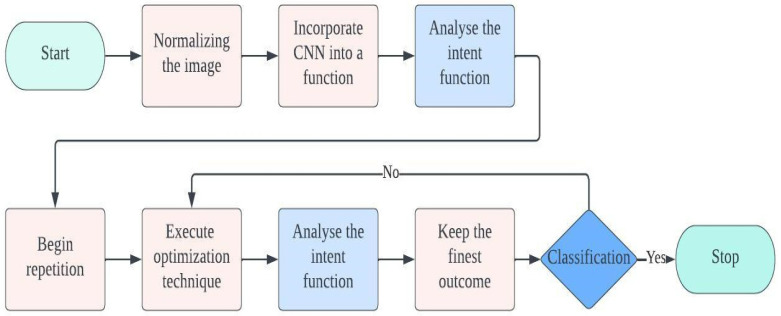
The typical metaheuristic algorithm-based hyperparameter optimization in a convolutional neural network block diagram.

The layers in a convolutional neural network (CNN) are structured into feature maps, adhering to the principles of local connectivity and weight distribution. Each individual neuron in a feature map establishes connections with localized patches in the preceding layer using a set of weights called a filter bank. Within feature maps, all units share a common filter row, but each feature map utilizes distinct filter banks. This approach aims to leverage the interconnectedness of neighboring pixels to exploit the location-independent properties of local image features and reduce the number of parameters involved. The aggregated weighted inputs are then passed through an activation function, such as the sigmoid or rectified linear unit (ReLU). The activation function plays a crucial role in introducing non-linear modifications to the transmitted data, thereby enhancing the effectiveness of subsequent processing stages. The pooling layer comes after the convolutional layer and employs a technique called sub-sampling to merge data from the convolutional layer. The primary objective of this approach is to reduce the size of the image while preserving significant information. Two commonly used techniques in this layer are max pooling and mean pooling [32]. The classifier, located in the last layer of the convolutional neural network (CNN), determines the category of the input data by utilizing the features collected and learned by the CNN. The number of classes or classifications is directly related to the number of units in the fully connected (FC) layer. The effectiveness of a CNN model relies on the selection and configuration of its hyperparameters. Researchers propose that optimizing these hyperparameters is crucial for achieving exceptional outcomes and enhancing accuracy. Table 1 shows the hyperparameters of the CNN architecture along with their descriptions, as previously mentioned in Section 1. Metaheuristic algorithms are widely recognized as effective techniques for improving the performance of CNN architectures through hyperparameter optimization. Figure 3 illustrates the application of an optimization methodology to enhance the hyperparameters of a CNN. The optimization process begins with initializing the population within the metaheuristic algorithm. The number of hyperparameters directly influences the dimensions subject to optimization. Prior to inputting images into the CNN, they undergo a normalization process. The recommended approach involves encapsulating the CNN architecture behind a function, allowing for its subsequent invocation during the evaluation of the fitness function, where hyperparameter optimization takes place. After constructing the fitness function, the iterations in the metaheuristic algorithm are adjusted based on the specific method being used. The optimal solution is selected after careful evaluation of the various options. The termination criteria are evaluated at the end of the process, and if not met, the method continues exploring alternative options until the criteria are satisfied.

### 2.4. Transfer Learning Technique

Transfer learning approaches are used to improve the performance of deep learning models when dealing with limited datasets, such as medical images. Training deep learning models from scratch often requires a large amount of data, computational resources, and time. To address these challenges, researchers employ pre-trained models [33]. These models have learned significant features from large datasets in various deep learning frameworks. Transfer learning allows deep learning systems to effectively learn from smaller datasets by leveraging the acquired representations. Pre-trained networks are widely used for small datasets, such as ImageNet, which is commonly utilized in various applications. Several pre-trained models, including GoogLeNet, AlexNet, VGG16, VGG19, ResNet, Inception, and DenseNet, have been trained using the ImageNet dataset. Transfer learning involves various strategies, such as feature extraction and augmentation techniques. In the feature extraction method, the convolutional component, which includes convolution and pooling layers, is retained in the network architecture. These layers act as a static feature extractor and are borrowed from a pre-trained network developed on the ImageNet dataset. After selecting a feature extractor, a classifier may be applied to the data. Additionally, fine-tuning requires a new set of fully connected layers to be added to an existing model and then retrained using the input data. The convolutional component of the pre-trained model is optimized with the help of the back-propagation method. The fine-tuning technique, like the feature extraction approach, requires unfreezing the last layers of the frozen convolutional area. Both the layers saved from the feature extractor and the newly added classifier need to be retrained. The purpose of fine-tuning is to increase the specificity of the previously trained model for the current task at hand [34].

### 2.5. Enhanced Ant Colony Algorithm

This section presents the proposed enhanced Ant Colony Optimization (EACO) approach. Evaluation of the initial EACO’s performance reveals that it does not thoroughly explore all possibilities within the search space. Moreover, it exhibits a poor convergence rate due to its division of the optimization stages into three distinct parts. To improve upon the original Ant Colony Optimization (ACO) and utilize it for optimizing the hyperparameters of the pre-trained CNN framework, the opposition-based learning (OBL) technique is employed. The EACO’s pseudo-code is presented in Algorithm 1, and to enhance the search operation, the startup phase of the search procedure used the OBL approach to increase the ACO’s selection as follows:

(8) $$OPS={ia}_{b}+va_{b}-x_{a},\;a\in 1,\;2,\;3,\;\ldots ,\;M_{n}$$

where OPS is a vector produced by applying OBL${ia}_{b}$, and $va_{b}$ are lower and higher region of the *b*th component of X, respectively. The different stages of the proposed EACO are described in the following subsections.

#### 2.5.1. Process of Establishment, Enhancement, and Culmination of EACO

The EACO begins by setting its parameters: maximum number of iterations $i_{max}$, size of the population $M_{n}$, ant aggregating devices (AAD), and the measurement *Measure*. The ACO starts by launching the initial search both x_0 and the outcome is saved. The OBL technique is then used to calculate the OPS of the starting population using Equation (8). As seen in Section 2.1, the optimization procedure is separated into four steps. After these phases have been completed, the OBL method is employed to determine the function of fitness for each solution in *x* and $\bar{x}$, and the suggested approach updates the global best solution by calculating and comparing the fitness of $x_{a}$ and OPS. After completing the optimization procedure, the memory is saved and the AADs are computed.

#### 2.5.2. Complex computations of EACO

This part explains the time and space expenditures of EACO as followed,

(i) Complexity of time: The EACO generates $M_{n}$ search bot with the measurement Measure, and the initialization time complexity was $T\left(M_{n}\times Measure\right)$. In addition, the EACO calculates every search bot’s fitness as $T\left({Ii}_{max}\times M_{n}\times Measure\right)$, where ${Ii}_{max}$ predicts the maximum level of iterations. In addition, the EACO expects to perform $T_{t}$ number of its primary processes. Hence, the time complexity of the EACO is illustrated by $T\left(M_{n}\times Measure\right).$
(ii) Complexity of space: The EACO space complexity is represented by $T\left(M_{n}\times Measure\right).$
**Algorithm 1.** EACO pseudo-code computational algorithm**Input:** ${Ii}_{max}$ = 40; $M_{n}$ = 25; P = 0.4, AAD’s = 0.1; down bound (db), up-bound (ub), measurement $M_{easure}$.
**Outcome**: To determine the better position and fitness in search space, and it can be initialized the population $x_{i}^{0}$ randomly along with the measurement $M_{easure}$.
However, the OBL need to apply on the population (initial) $x_{i}^{0}$ by Equation (8), and the outcomes are saved in OPS.
**While** $i\;\leq \;{Ii}_{max}$ **do**
**for** $j\;\leq M_{n}$ **do** Compute $x_{j}$ deploying the function (fitness) and save outcome in ${fit}_{j}$.
Evaluate the value of fitness.
**if** ${Fit}_{j}<Fit\;{OPS}_{j}$ **then** $x_{j}={OPS}_{j};$
**end if**
**end for**
Activate the memory storing.
**if** $i<\frac{Ii_{max}}{3}$ then
Update memory by Equation (4).
**else if** $\frac{Ii_{max}}{3}<i<2\times \frac{Ii_{max}}{3}$ then
Update the first and second half of the solution by using Equations (5) and (6), respectively.
**end if**
Perform memory store and update.
Apply AAD.
Save all.
**end while**
Return the better outcome.

## 3. Proposed Framework EACO–ResNet101 Classification

**Figure 4:**
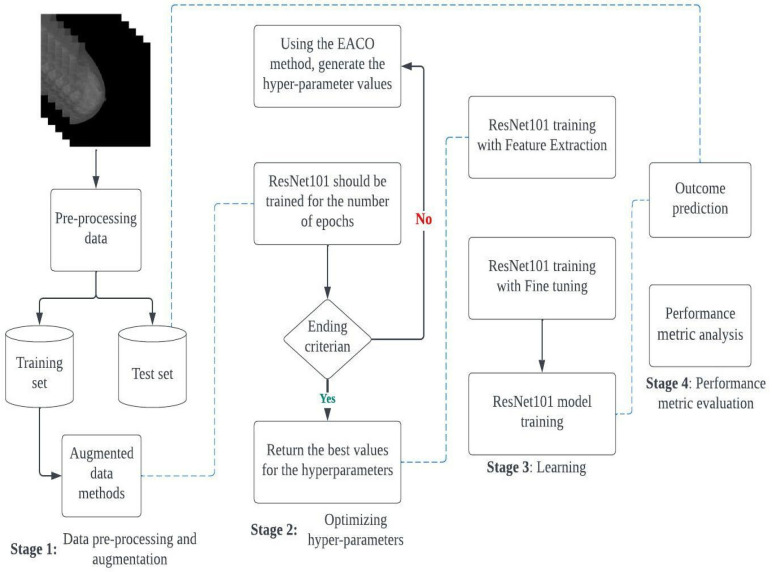
Stages of the proposed Enhanced Ant Colony Optimization–ResNet101 architecture block diagram.

In this section, we introduce the proposed framework, EACO–ResNet101, which incorporates transfer learning from a pre-trained convolutional neural network (CNN) model. The specific pre-trained model utilized in this study is ResNet101. The primary objective of this research is to improve the performance of the pre-trained CNN model by employing the Enhanced Ant Colony Optimization (EACO) method to optimize its hyperparameters. Once the best parameter values are identified, the ResNet101 model undergoes training using transfer learning techniques. To evaluate the performance of the model, a separate test set is used for verification. Additionally, the trained model is subjected to validation using the test set. The proposed framework is structured into four distinct stages, as depicted in Figure 4.

The order of the different stages is as follows: (i) Stage 1: Pre-processing and augmentation; (ii) Stage 2: Hyperparameter optimization; (iii) Stage 3: Model training; (iv) Stage 4: Performance metric evaluation. In the first stage, the datasets were enhanced and divided into two sets for training and testing. Various data augmentation techniques were also employed to expand the training sets. The proposed model was tested on two datasets: CBIS-DDSM and MIAS. During the second stage, EACO was applied to optimize the hyperparameters of the pre-trained CNN architecture (ResNet101). The hyperparameter values generated in the second stage were then used to train ResNet101 entirely in the third stage. This facilitated the architecture in effectively diagnosing the test set in the subsequent stage. The following sections will provide a detailed explanation of the different stages of EACO–ResNet101.

### 3.1. Stage 1: Pre-Processing and Augmentation

The two mammographic datasets were subjected to data pre-processing and augmentation at this stage. Before performing data augmentation, the images were upgraded by eliminating noise and scaling them to 224 × 224 resolution, lowering storage space and processing time [35]. Furthermore, numerous data augmentation approaches were used to enhance training sets, reduce overfitting, accelerate convergence, and improve standardization. To expand the images in the dataset’s training set, data augmentation was carried out using the Keras ImageDataGenerator. Table 2 shows the range of data augmentation techniques employed.

### 3.2. Stage 2: Optimizing Hyperparameters

Using a pre-trained model’s framework while making minor adjustments is what transfer learning is all about. Changing the classifier is a major adjustment that often necessitates changing or establishing new hyperparameter values. The classification performance of a convolutional neural network (CNN) is very sensitive to its hyperparameter setup. The suggested EACO–ResNet101 model seeks to optimize eight hyperparameters, as detailed in Section 2.3. The number of units in the first three dense layers, as well as the learning rate, batch size, and dropout rates of three distinct dropout layers, are all examples of such hyperparameters. The model’s capability for learning and generalization depends on the values of its many hyperparameters. The EACO–ResNet101 model’s goal is to improve the CNN’s efficiency and precision by adjusting its hyperparameters. When there are eight individual hyperparameters that need to be fine-tuned, the search space has eight dimensions, with each dimension representing a different possible setting for the hyperparameters. The Enhanced Ant Colony Optimization (EACO) technique is used to effectively search this high-dimensional space for the optimal settings for the ResNet101 hyperparameters.

### 3.3. Stage 3: Model Learning

In this study, the ResNet101 model is used for learning purposes with the available datasets, namely CBIS-DDSM and MIAS. The learning process involves a combination of feature extraction and modification techniques. The feature extraction strategy entails keeping the convolutional base of ResNet101 unchanged while replacing the outdated classifier with a more suitable one for the specific datasets. In addition to the thick layers and the flattened layer, the newly developed classifier has three dropout layers in between. Dropout rates for all layers are optimized, the percentage of neurons utilizing the ReLU activation function in the first three dense layers is optimized, and the ideal learning rate for the convolutional layer is optimized using the EACO approach. The softmax function, used by a neuron in the last dense layer, is crucial for classification. To achieve customization, the final two blocks of the convolutional part of ResNet101 are re-trained while simultaneously incorporating the new classifier. This customization process occurs once the newly trained classifier has undergone several epochs to ensure its convergence and stability. By employing a mix of feature extraction and modification techniques, along with the optimization of hyperparameters using the EACO method, the ResNet101 model is fine-tuned and tailored to the specific characteristics of the CBIS-DDSM and MIAS datasets. This process aims to enhance the model’s performance and accuracy for image recognition and classification tasks related to medical imaging.

### 3.4. Stage 4: Performance Metric Evaluation

Measures such as accuracy, sensitivity, specificity, precision, F-score, and area under the curve (AUC) were employed throughout this investigation to determine how well the proposed strategy worked. Accuracy (Acc) is defined as the proportion of correctly labeled instances. Analysis of sensitivity (Se) reveals how many true positives there are out of the total number of instances. Specificity (Sp) reflects the accuracy of both normal predictions and overall pessimistic predictions. Precision (Pr) measures the reliability of atypical breast cancer predictions. The average F-score assesses the accuracy of the test. AUC provides insights into the model’s performance in different settings.

(9) $$\mathrm{Accuracy}\;\mathrm{metric}\;(\mathrm{Acc})=\frac{\alpha +\beta }{\alpha +\beta +\gamma +\delta }$$

(10) $$\mathrm{Sensitivity}\;(\mathrm{Se})=\frac{\alpha }{\alpha +\delta }$$

(11) $$\mathrm{Specificity}\;(\mathrm{Sp})=\frac{\beta }{\beta +\gamma }$$

(12) $$\mathrm{Precision}\;(\mathrm{Pr})=\frac{\alpha }{\alpha +\gamma }$$

(13) $${F1}_{i}=\frac{\alpha_{i}}{\alpha_{i}+\gamma_{i}}$$

(14) $$F1\;\mathrm{score}=\frac{1}{r}\sum_{i=1}^{r}F1_{i}$$

(15) $$\mathrm{AUC}=\frac{\sum Q_{j}\left(J_{t}\right)-J_{t}(J_{t}+1)/2}{J_{t}+J_{i}}$$

where *α*—true positive, *β*—true negative, *γ*—false positive, *δ*—false negative, $J_{t}$ and $J_{i}$ represent the positive and negative images, respectively, and $Q_{j}$—rate of the $j_{th}$ positive image.

## 4. Experimental Results and Metric Analysis

The performance of the proposed EACO–ResNet101 model in classifying breast cancer detected by mammography is validated through the results described and analyzed in this section. The section is organized as follows: Section 4.1 presents the datasets used in this study. Section 4.2 introduces the framework proposed in this paper. The experimental settings for the EACO parameters are presented in Section 4.3. The outcomes of the CBIS-DDSM dataset are discussed in Section 4.4, while the outcomes of the MIAS dataset are described in Section 4.5. The proposed EACO–ResNet101 model is compared to four additional metaheuristic algorithms that are compatible with the ResNet101 architecture: HHO–ResNet101, MPA–ResNet101, GSA–ResNet101, and WOA–ResNet101.

### 4.1. Materials (Dataset Usage)

The proposed framework in this research was evaluated using two datasets, MIAS and CBIS-DDSM, which are each described below.

#### 4.1.1. CBIS-DDSM

CBIS-DDSM is a superior replacement for the DDSM mammography dataset. After being decompressed, its pictures may be viewed in DICOM format on a computer. We followed the CBIS-DDSM recommendations for processing this dataset and converting the DICOM format into PNG files to train a strategy for classifying pictures as benign or malignant. The proposed framework was trained and evaluated on a total of 5482 images [36]. The dataset’s description and sample count for the training and test sets are shown in Table 3.

#### 4.1.2. MIAS

The 344 mammography images in the MIAS dataset have a 1024 × 1024 resolution. There are two classes in this dataset: 224 (65%) normal and 120 (35%) abnormal. Two types of abnormal images were created: benign, which contains 65 images, and malignant, which has 48 images. The MIAS dataset offers essential information for each class [37].

**Figure 5:**
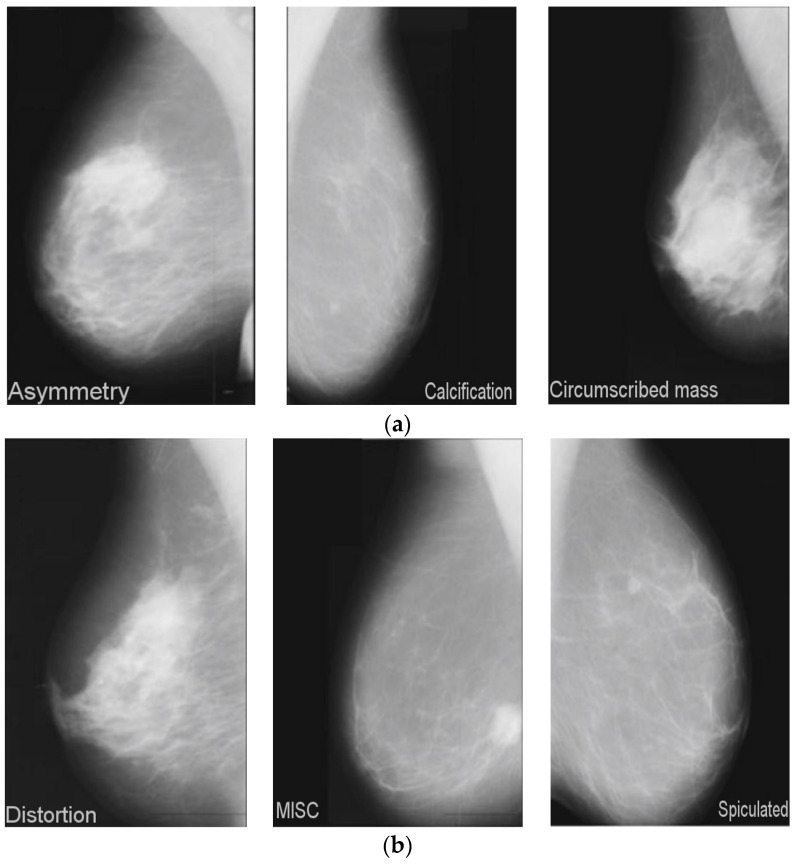
MIAS dataset category (**a**) asymmetry, calcification, circumscribed mass images; (**b**) distortion, MISC, spiculated images.

The data identifies the type of anomaly, such as calcifications, masses, and asymmetries. Six categories are used to classify this dataset, as illustrated in Figure 1. The number of images increases (4%) after using the data augmentation methods described in Section 4.1 on the MIAS dataset, as seen in Table 4. Figure 5 depicts the detailed image categorization of the MIAS dataset.

### 4.2. Hyperparameter Setting

Table 5 includes a list of the parameters used in the proposed framework. The population size is 30 and the highest possible number of iterations is 50, which is nearly equivalent to the number of dimensions we have. The initial learning rate parameter is optimized by the EACO algorithm to fit in the ideal region. The possible values of the learning rate are 1 × 10^−7^, 1 × 10^−5^, and 1 × 10^−3^, and it needs to be a low value in the fine-tuning technique because the number of model modifications must be kept to a minimum to prevent losing the features obtained by the feature extraction approach.

The number of neurons can be any value between 25 and 100, and the range of acceptable dropout rates is between 0.2 and 0.8, so the optimal batch size is between 1 and 64, the dropout rates are between 0.2 and 0.8, the searching range of dropout rates is bounded by a lower bound of 0.2 and an upper bound of 0.8, and the batch size is between 1 and 64. After trying out several numbers, we settled on a training duration of 101 epochs for ResNet101.

The experiment showed that each EACO’s training procedure requires exponentially more time when using more than 30 epochs.

Additionally, ResNet101’s results were not reliable enough when using fewer than 30 epochs. Consequently, 30 epochs were used to train the ResNet101. The EACO dimension parameter represents the number of hyperparameters that the proposed EACO can optimize. The learning rate, batch size, three dropout rates of the three dropout layers, and the number of units of the initial three dense layers make up the eight hyperparameters.

### 4.3. EACO–ResNet101 Model Evaluation for the CBIS-DDSM Dataset

This subsection presents the outcomes of the proposed EACO–ResNet101 model using the hyperparameters calculated by the EACO based on the CBIS-DDSM dataset. It also provides a comparison with other related research and work. Additionally, to demonstrate the efficiency of the EACO in identifying the optimal values for the hyperparameters of the ResNet101 model, which lead to the highest accuracy, we compare it to the ResNet101 model configured manually.

**Figure 6:**
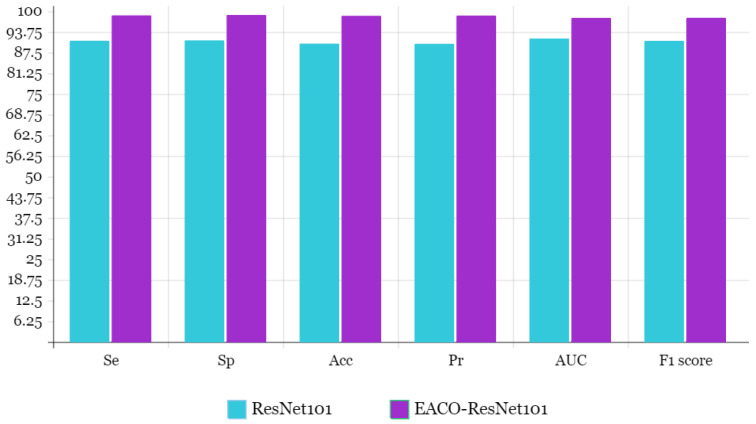
Bar chart representation of proposed and ResNet101 model outcome comparison.

Table 6 displays the accuracy, sensitivity, specificity, precision, F1-score, and AUC of the EACO–ResNet101 model on the CBIS-DDSM dataset. On the test set, the proposed technique obtained 98.63% accuracy. Se, Sp, Pr, F1-score, and AUC were all 98.76%, 98.89%, 98.71%, 98.04%, and 98.01% on average. Figure 6 depicts the graphical representation of the proposed system and ResNet101 outcome comparison.

Table 7 shows the proposed EACO–ResNet101 model with the ACO–ResNet101 technique based on manual searching of the CBIS-DDSM dataset. According to the results presented in Table 8, the proposed EACO–ResNet101 model outperforms the ResNet101 model without the hyperparameter optimization. Although the ResNet101 architecture’s accuracy is 90.21%, its sensitivity is 91.09%, its specificity is 91.20%, its precision is 90.16%, the F1 score is 91.06%, and the AUC is 91.76%.

**Figure 7:**
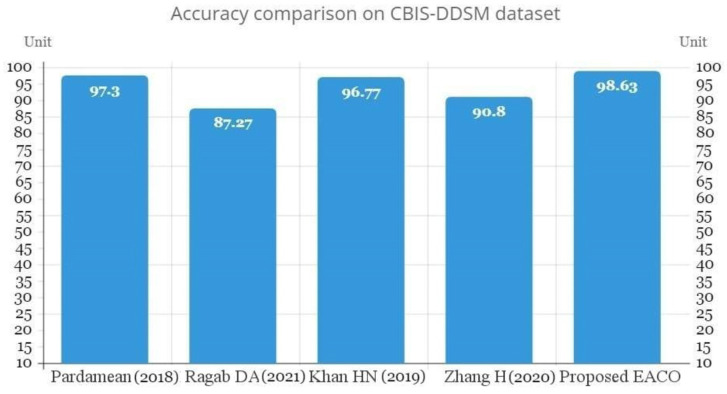
Accuracy comparison of proposed and state-of-the-art methods on CBIS-DDSM dataset [16,29,30,32].

According to the Acc, the suggested EACO–ResNet101 model improves on the ResNet101 architecture by 8.42%, Se is 7.67%, Sp is 7.69%, Pr is 8.55%, F1 score is 6.98%, and AUC is 6.25%. Additionally, using the CBIS-DDSM dataset, Table 8 compares the performance of the proposed EACO–ResNet101 model with state-of-the-art methods on breast cancer diagnosis. Figure 7 illustrates the accuracy outcome comparison of the proposed and existing methods.

### 4.4. EACO–ResNet101 Model Evaluation for the MIAS Dataset

This subsection presents the outcomes of the proposed method, EACO–ResNet101, utilizing the hyperparameters derived by the IMPA algorithm based on the MIAS dataset. The results are presented and compared with other similar investigations. Additionally, we compare it to the ResNet101 model with manually configured hyperparameters to demonstrate the effectiveness of the EACO in selecting the optimal values that lead to the highest accuracy.

Table 9 presents the evaluation results of EACO–ResNet101 for the MIAS dataset in terms of sensitivity, specificity, accuracy, precision, AUC, and F1-score. The proposed method achieved an accuracy of 99.15%. The average values for sensitivity, specificity, accuracy, AUC, and F1-score were 97.86%, 98.88%, 98.80%, 99.12%, and 97.60%, respectively. Table 10 provides a comparison between the EACO–ResNet101 model and the ACO–ResNet101 approach, which involves manually selecting hyperparameters for the MIAS dataset. The results in Table 10 demonstrate that the proposed ResNet101 model outperforms the ResNet101 architecture with non-optimized hyperparameters. The ResNet101 architecture achieved an accuracy of 87.67%, sensitivity of 86.98%, specificity of 89.12%, precision of 88.10%, F1-score of 87.32%, and AUC of 89.76%.

**Figure 8:**
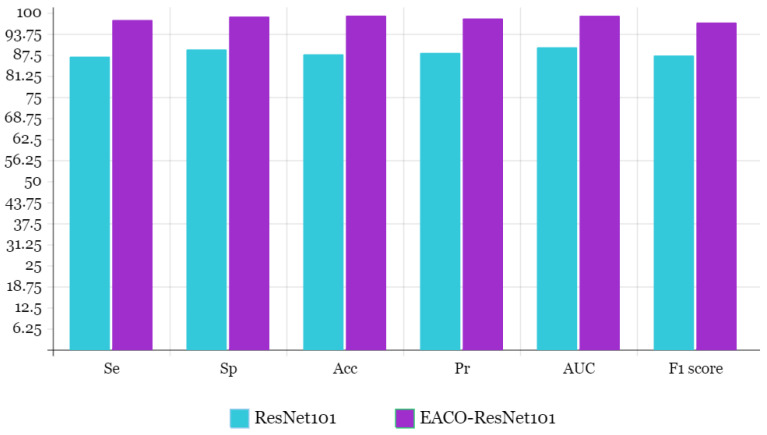
Bar chart representation of proposed and ResNet101 model outcome comparison.

The proposed EACO–ResNet101 model demonstrates superior performance compared to the ResNet101 architecture. It achieves an 11.48% higher accuracy, 10.88% higher sensitivity, 9.76% higher specificity, 10.2% higher precision, 9.78% higher F1 score, and 9.36% higher AUC. Figure 8 visually illustrates the comparison between the proposed model and ResNet101 on the MIAS dataset. Table 11 presents a performance comparison between the proposed EACO–ResNet101 model and state-of-the-art methods for breast cancer diagnosis on the MIAS dataset.

### 4.5. Evaluation of Various Optimization Techniques

This article conducts a comparative analysis of the EACO technique against other contemporary metaheuristic algorithms. The primary goal is to demonstrate the effectiveness of the EACO algorithm in accurately determining the optimal configurations for the hyperparameters of the ResNet101 architecture, leading to higher levels of accuracy in image recognition tasks. The study includes a comparison with four other widely recognized metaheuristic algorithms: the Gravitational Search Algorithm (GSA), the Harris Hawks Optimization (HHO) method, the Particle Swarm Optimization (PSO) algorithm, and the original Ant Colony Optimization (ACO) algorithm. These algorithms have gained significant attention in the field and are frequently cited in the relevant literature. By evaluating and comparing the performance of the EACO technique with these established algorithms, the research aims to demonstrate the superiority of EACO in hyperparameter optimization for deep learning models like ResNet101. This comparative analysis will provide valuable insights into the strengths and weaknesses of each algorithm, ultimately contributing to the advancement of optimization techniques in the field of deep learning and image recognition.

You can refer to the GSA–ResNet50, HHO–ResNet101, PSO–ResNet50, and ACO–ResNet101 models by their respective algorithmic names. These models are classified as hybrids since they use the ResNet structure. All examined algorithms are constructed with the same parameters, as indicated in Table 5, to guarantee a fair and comprehensive evaluation of the EACO algorithm compared to others. According to Table 12, in the classification of mammography breast cancer datasets, the EACO–ResNet101 model outperforms the ACO–ResNet50, GSA–ResNet50, HHO–ResNet101, and PSO–ResNet50 models. When applied to ResNet101, the EACO approach optimizes the network’s hyperparameters, resulting in higher accuracy. Particularly, the EACO–ResNet101 model clearly outperforms the original ACO algorithm in fine-tuning ResNet101’s hyperparameters, as demonstrated by its superior performance over the ACO–ResNet101 model. The ACO–ResNet101 model achieves 96% accuracy on the CBIS-DDSM dataset and 94.7% accuracy on the MIAS dataset. On the other hand, the EACO–ResNet101 model outperforms the other tested models by a significant margin. The GSA method achieves a 95.5% accuracy rate on the CBIS-DDSM dataset and a 94.5% accuracy rate on the MIAS dataset. The HHO method obtains an accuracy of 94.7% on the CBIS-DDSM dataset and 94.8% on the MIAS dataset. The Particle Swarm Optimization (PSO) method achieves an accuracy of up to 95.1% on the CBIS-DDSM dataset and 93.5% on the MIAS dataset. Overall, the EACO–ResNet101 model outperforms the other models in correctly classifying mammography breast cancer datasets. When applied to medical image classification tasks, the EACO algorithm is shown to be an efficient and effective way of optimizing hyperparameters and improving the accuracy of the ResNet101 architecture.

## 5. Conclusions and Future Work

Deep learning has become a crucial approach in the field of medical imaging classification. Convolutional neural networks (CNNs) are extensively used in biomedical image classification to automatically extract features. However, each layer of a CNN requires its own set of hyperparameters, making the fine-tuning process essential for achieving optimal performance in classification tasks. Manually selecting hyperparameters can be challenging and time-consuming, making it impractical for obtaining ideal results. To address this issue, metaheuristic strategies have been widely adopted for hyperparameter optimization in various domains. This paper introduces a novel breast cancer classification model that combines a pre-trained CNN architecture, ResNet101, with an enhanced metaheuristic optimization strategy. The proposed method utilizes the opposition-based learning (OBL) strategy in the ant colony algorithm, a popular optimization approach, to enhance performance by promoting exploitation and reducing the risk of converging to local optima. The hyperparameters for the ResNet101 architecture are optimized using the Enhanced Ant Colony Optimization (EACO) technique, resulting in the development of the EACO–ResNet101 model. By integrating advanced optimization methods with a powerful CNN architecture, the EACO–ResNet101 model aims to achieve superior performance in breast cancer classification tasks. This combination of techniques represents a promising approach to enhancing the accuracy and efficiency of medical image classification models.

This study represents the first effort in utilizing the EACO algorithm to optimize the hyperparameters of the ResNet101 architecture for breast cancer classification. The suggested model is extensively compared to current state-of-the-art methods and approaches in the field of convolutional neural networks (CNNs). The comparative findings demonstrate that the suggested model is superior in detecting breast cancer. The evaluation is performed using two mammography datasets, the Mammographic Image Analysis Society (MIAS) dataset, and the selected breast imaging subset of the Digital Database for Screening Mammography (CBIS-DDSM). The suggested model will be tested again in the future using larger datasets with more images. Additionally, several pre-trained models, including DenseNet201, DenseNet121, and Inception, will be incorporated into the breast cancer classification process, utilizing a variety of feature extraction strategies to enhance classification accuracy. Optimal management of the interplay between data augmentation, dataset variety, and extensive review is crucial for developing trustworthy AI systems for medical diagnosis. The ongoing model development and improvement aim to increase accuracy and reliability in breast cancer classification, leading to improved medical diagnosis and therapy.

## Figures and Tables

**Table 1:** CNN’s hyperparameter interpretation.

Name of the Hyperparameter	Interpretation
Progress of learning	One of the important hyperparameters that has a major impact on output performance is the CNN architecture’s initial learning rate. The model requires more iterations when the learning rate is low.
Summary of units in a hidden layer	Although adding more hidden layer units improves the model, it decreases the speed of computation.
Size of the batch	The number of sub-samples transmitted to the network for parameter updates is referred to by this phrase.
Rate of retention	By improving validation accuracy and subsequently generalizing, a dropout is a regularization strategy that lowers overfitting.
Role of activation	Deep learning approaches can learn nonlinear prediction limitations through the use of activation functions.
Length of epoch	It refers to how frequently the full training data set is run through the learning cycle.

**Table 2:** The techniques for data augmentation and their boundaries.

Data Augmentation Method	Boundary
Shedding	0.11
Expanding	0.13
Spin	15
Height change	0.32
Width change	0.32
Horizontal rotation	True
Feature set position	True
Vertical rotation	True
Complete mode	Reflect

**Table 3:** CBIS-DDSM dataset description.

Dataset Name	Total Images	Type	Training Images (70%)	Testing Images (30%)
CBIS-DDSM	5482	Normal	1898	803
		Malignant	1939	843

**Table 4:** MIAS dataset description.

Dataset Name	Total Images	Category		Training Images (70%)	Testing Images (30%)
MIAS	1376	Normal	894	962	414
		Malignant	482		

**Table 5:** EACO hyperparameter setting.

Parameters	Values
Maximum number of iterations	45
Size of the population	35
Dimension	8
Rate of learning	(1 × 10^−7^, 1 × 10^−5^, 1 × 10^−3^)
Size of the batch	(64, 1)
Rate of dropout	(0.2, 0.8)
Neurons count	(25, 50, 75, 100)
ResNet101 training epoch	30

**Table 6:** Proposed system parameters outcome based on CBIS-DDSM dataset.

Model	Parameter Metrics					
	Se (%)	Sp (%)	Acc (%)	Pr (%)	AUC (%)	F1 Score (%)
EACO–ResNet101	98.76	98.89	98.63	98.71	98.01	98.04

**Table 7:** Proposed system parameters outcome versus ResNet101 based on CBIS-DDSM dataset.

Models	Parameter Metrics					
	Se (%)	Sp (%)	Acc (%)	Pr (%)	AUC (%)	F1 Score (%)
ResNet101	91.09	91.2	90.21	90.16	91.76	91.06
EACO–ResNet101	98.76	98.89	98.63	98.71	98.01	98.04
Augmentation (%)	7.67	7.69	8.42	8.55	6.25	6.98

**Table 8:** Outcome comparison of the EACO–ResNet101 and other state-of-the-art methods on the CBIS-DDSM dataset.

Author	Images	Dataset	Model	Se (%)	Sp (%)	Acc (%)	Pr (%)	AUC (%)	F1 Score (%)
Pardamean [16]	2398	DDSM	CNN-Yolo	93.21	94.12	97.3	96.4	97.1	N/A
Ragab DA [29]	5270	CBIS-DDSM	ResNet101	86.1	89.14	87.27	86.72	95.04	N/A
Khan HN [30]	3570	CBIS-DDSM	ResNet101	93	88.64	96.77	95.12	93.5	94.32
Zhang H [32]	2832	CBIS-DDSM	AdaBoost	82.98	98.3	90.8	86.1	98.4	N/A
Proposed EACO	5482	CBIS-DDSM	EACO–ResNet101	98.76	98.89	98.63	98.71	98.01	98.04

**Table 9:** Proposed system parameters outcome based on MIAS dataset.

Model	Parameter Metrics					
	Se (%)	Sp (%)	Acc (%)	Pr (%)	AUC (%)	F1 Score (%)
EACO–ResNet101	97.86	98.88	99.15	98.3	99.12	97.6

**Table 10:** Proposed system parameters outcome versus ResNet101 based on MIAS dataset.

Models	Parameter Metrics					
	Se (%)	Sp (%)	Acc (%)	Pr (%)	AUC (%)	F1 Score (%)
ResNet101	86.98	89.12	87.67	88.1	89.76	87.32
EACO–ResNet101	97.86	98.88	99.15	98.3	99.12	97.1
Augmentation (%)	10.88	9.76	11.48	10.2	9.36	9.78

**Table 11:** Outcome comparison of the EACO–ResNet101 and other state-of-the-art methods on the MIAS dataset.

Author	Images	Dataset	Model	Se (%)	Sp (%)	Acc (%)	Pr (%)	AUC (%)	F1 Score (%)
Xiang Yu [32]	340	MIAS	DenseNet201	94.6	91.71	92.81	91.63	N/A	N/A
Ragab DA [29]	1292	MIAS	DCNN	96.7	92.3	95.5	94.7	98.9	N/A
Tan YJ [31]	332	MIAS	CNN	82.8	82.7	82.71	81.8	N/A	N/A
Khan [30]	332	MIAS	CNN	90.8	90.51	89.51	89.8	N/A	N/A
Proposed EACO	5482	MIAS	EACO–ResNet101	97.86	98.88	99.15	98.3	99.12	97.1

**Table 12:** Metric comparison of proposed and other state-of-the-art method algorithms.

Model	Dataset	Se (%)	Sp (%)	Acc (%)	Pr (%)	F Score (%)
ACO–ResNet101	CBIS-DDSM	93.4	95.3	96	95.1	94.1
GSA–ResNet50		94.3	95.2	95.5	95.2	94.3
HHO–ResNet101		94.1	95	94.7	94.3	94.7
PSO–ResNet50		93.3	94.9	95.1	94.4	94.6
EACO–ResNet101		98.7	98.8	98.6	98.7	98.04
ACO–ResNet101	MIAS	94.7	94.3	95.1	94.4	95.01
GSA–ResNet50		94.03	93.4	94.5	94.3	94.1
HHO–ResNet101		93.7	94.3	94.8	93.8	94.1
PSO–ResNet50		93.2	93.3	93.5	93.1	93.06
EACO–ResNet101		97.86	98.88	99.15	98.3	97.1

## Data Availability

Not applicable.
